# TIPE2 Inhibits Lung Cancer Growth Attributing to Promotion of Apoptosis by Regulating Some Apoptotic Molecules Expression

**DOI:** 10.1371/journal.pone.0126176

**Published:** 2015-05-06

**Authors:** Qing-Qing Liu, Feng-Feng Zhang, Fang Wang, Jing-Hua Qiu, Chun-Hua Luo, Guo-Yong Zhu, Ying-Fu Liu

**Affiliations:** 1 Department of Basic Medical Sciences, Medical College, Xiamen University, Xiamen, Fujian, PR China; 2 The Department of Pathology, The Traditional Chinese Medical Hospital of Xiamen, Xiamen, Fujian, People’s Republic of China; 3 Department of Thoracic Surgery, The First Affiliated Hospital of Xiamen University, Xiamen, Fujian, PR China; 4 Department of Basic Medicine Science, NanYang Medical College, Nanyang, China; H.Lee Moffitt Cancer Center & Research Institute, UNITED STATES

## Abstract

Recent studies found that TIPE2 was involved in cancer development. However, little is known about TIPE2 in lung cancer. Our study aims to clarify the role of TIPE2 in lung carcinogenesis. We examined the expression of TIPE2 in lung squamous cancer (LSC), small cell lung cancer and lung adenocarcinoma (AdC) tissues and found that TIPE2 expression was lost in small cell lung cancer, compared with adjacent non-tumor tissues. Overexpression of TIPE2 significantly inhibited the growth of lung cancer cell H446 *in vitro* and even suppressed tumor formation *in vivo*. Flow cytometry analysis found TIPE2 overexpression promoted apoptosis of H446. In TIPE2 over-expression cells, caspase-3, caspase-9, and Bax were significantly up-regulated while Bcl-2 was down-regulated. Moreover, coincident results were shown by immunohistochemistry in tumors from nude mice. TIPE2 inhibited the phosphorylation of Akt, while promoting the phosphorylation of P38, but had no effect on IκBα and ERK pathway. Taken together, TIPE2 promoted lung cancer cell apoptosis through affecting apoptosis-related molecules caspase-3, caspase-9, Bcl-2 and Bax, possibly via regulating P38 and Akt pathways, indicating that TIPE2 might be a novel marker for lung cancer diagnosis and therapy.

## Introduction

Lung cancer is one of the most common cancers worldwide and continues to be the leading cause of cancer-related mortality [1]. There are approximately 1000,000 new lung cancer cases and more than 800,000 estimated deaths every year in the world [2]. Despite recent advances in diagnostic and therapeutic techniques, the 5-year overall survival rate of lung cancer patients is still less than 15% [3]. Therefore, it is an urgent need to reveal the molecular mechanism of lung cancer and find novel candidate biomarkers with potential clinical value, which will facilitate the development of more effective therapeutic targets and treatment strategies.

Tumor necrosis factor (TNF)-alpha-induced protein 8-like 2 (TNFAIP8L2), also known as TIPE2, is a member of the TNFAIP8 family and a newly described negative regulator of both innate and adaptive immunity [4]. It prevents hyper-responsiveness and maintains immune homeostasis via suppressing TLR and TCR function, and its deficiency in mice leads to multi-organ inflammation [4]. Moreover, in patients with chronic inflammatory diseases such as systemic lupus erythematosus and hepatitis, TIPE2 expression is also down-regulated and inversely correlates with disease progression [5,6]. However, different from murine TIPE2, which is preferentially expressed in hematopoietic cells, human TIPE2 is also expressed in a wide variety of non-hematopoietic cell types [4,7,8].

Human TIPE2 shares approximately 53% identity and 78% similarity amino acid sequence with TNFAIP8, and about 94% homologous with murine TIPE2 [4]. Initial studies showed that other members of TIPE family such as TNFAIP8 and TIPE1 play roles in carcinogenesis in most human cancers [9–11]. The high-resolution crystal structure of TIPE2 reveals that it contains a large hydrophobic central cavity, which appears to be a DED-like domain differs from caspase-8 or cFLIP [12]. Murine TIPE2 inhibits MAPKs and NFκB signaling pathways which promote Fas-induced apoptosis [4]. Additionally, overexpression of TIPE2 in mice results in cell death and significantly inhibits Ras-induced tumorigenesis [13]. However, TIPE2 is not detectable or poorly expressed in most human carcinoma cell lines [7]. These results suggest that in addition to inflammation, TIPE2 may also be involved in cancer development.

Increasing studies have been focused on TIPE2 and human cancers in recent years. TIPE2 suppresses TLR4-mediated development of colon cancer by inhibiting caspase-8 activity [14], while its expression is positively correlated with TNM staging in renal cell carcinoma (RCC) patients [15]. Recently, TIPE2 is proved to be an endogenous inhibitor of Rac1 in liver by which it suppressed invasion and metastasis of hepatocellular carcinoma (HCC) [16]. However, the role of TIPE2 in lung cancer is unclear. In present study, we provide evidences firstly indicate that TIPE2 suppresses the growth and promotes apoptosis of lung cancer through regulating the Bcl-2/Bax balance and then leads to the activation of caspase-3 and caspase-9 possibly via affecting P38 and Akt pathway.

## Materials and Methods

### Ethics statement

The study was approval by the Institutional Research Ethics Committees of The First Affiliated Hospital of Xiamen University, and written informed consent was obtained from all patients. All specimens were handled and made anonymous according to the ethical and legal standards. All animal procedures in this study were approved by the Animal Experimentation Ethics Committee of Xiamen University.

### Tissue samples

The paraffin-embedded lung cancer specimens were used for immunohistochemical analysis. The paraffin-embedded lung cancer specimens consisted of 25 lung squamous cancer specimens, 20 lung adenocarcinoma specimens, 15 small lung cancer specimens and 30 adjacent non-tumor lung specimens. All the patients recruited in this study received neither chemotherapy nor radiotherapy before the surgery.

### Cell culture, plasmid construction and transfection

The human NCI-H446 cell lines were purchased from the Chinese Academy of Medical Sciences (Shanghai, China). The H446 cell was cultured in RPMI 1640 medium (Gibco) supplemented with 10% FBS (HyClone), 100 units/ml penicillin and 100 units/ml streptomycin. Cells were maintained in a humidified atmosphere with 5% CO_2_ at 37°C. Full-length human TIPE2 gene was generated from the cDNA clone by PCR and then cloned into the pRK5 vector by standard molecular techniques and verified by sequencing. Transfection of tumor cells with vector was performed using FuGENE HD Transfection Reagent (Promega) according to the manufacturer’s protocol. To generate stable transfected cell lines, transfected cells were selected in RPMI 1640 medium containing 500 μg/ml G418 for two weeks to isolate the formed colonies for further expansion. At the end of the transfection, TIPE2 expression was verified by Western Blotting.

### Immunohistochemistry (IHC)

Paraffin-embedded specimens (4 μm) were used for analysis as the standard immunohistochemical technique. Briefly, sections were incubated with anti-TIPE2 antibody (Abnova) overnight at 4°C. Secondary staining was performed with HRP-conjugated secondary antibody using a Max Vision kit. Immunoreactivity was visualized using a DAB Peroxidase Substrate kit (Maixin Co, Fuzhou, China) and counterstained with hematoxylin. In negative controls, primary antibodies were replaced by PBS. All IHC staining was independently evaluated by two experienced pathologists in an effort to provide a consensus on staining patterns according to a scoring method, as described previously [17,18]. At least 10 high-power fields, selected randomly, and >1000 cells were counted for each specimen. Each case was scored according to the intensity and area of staining. The intensity of staining was graded on the following scales: 0, no staining; 1+, mild staining; 2+, moderate staining; 3+, intense staining. The area of staining was evaluated as follows: 0, no staining of cells in any microscopic fields; 1+, <30% of tissue stained positive; 2+, 30–60% stained positive; 3+, >60% stained positive. A combined staining score (intensity + extension) of between 0 and 3 was considered to be low expression, whereas a score between 4 and 6 was considered to be high expression.

### Western blotting

Cells were harvested and lysed in lysis buffer with proteinase inhibitor cocktail (Roche) and PMSF (Sigma). Then 30 μg lysates from each sample was separated by 10% SDS-PAGE and transferred to PVDF membranes. Membranes were blocked with 5% non-fat milk and probed overnight at 4°C with the following primary antibodies: anti-TIPE2 (dilution, 1:800; Abnova), anti-caspase-3 p17 (dilution, 1:400; Santa Cruz), anti-caspase-9 (dilution, 1:400; Santa Cruz), anti-Bcl-2 (dilution, 1:200; Santa Cruz), anti-Bax (dilution, 1:1000; Santa Cruz), anti-ERK (dilution, 1:1000; CST), anti-phospho-ERK (dilution, 1:1000; R&D), anti-P38 (dilution, 1:1000; CST), anti-phospho-P38 (dilution, 1:1000; CST), anti-Akt (dilution, 1:1000; CST), anti-phospho-Akt (dilution, 1:1000; CST), anti-IκBα (dilution, 1:1000; CST), anti-phospho-IκBα (dilution, 1:1000; CST), and anti-β-actin antibody (dilution, 1:4000; Abcam), then followed by horseradish peroxidase-conjugated secondary antibodies (anti rabbit or mouse IgG, 1:5000; Santa Cruz) for 1 hour at room temperature. After washing, the blots were developed using ECL detection reagent (GE Healthcare) and quantitated by densitometry using ImageQuant image analysis system (Storm Optical Scanner). All the experiments were performed independently three times at least.

### Colony formation assay

Cells were seeded in six-well plates at a density of 500 cells per well, and replaced the medium every 3 days. Two weeks later, cells were washed by PBS, and fixed with methanol for 15 minutes, then stained with crystal violet. Colonies which consisted of more than 50 cells were counted and calculated as a percentage of that of the control group. The experiment was performed independently three times at least.

### MTT assay

Cell viability was detected by MTT assay. A total of 3000 cells per well were seeded in 96-well plates with three triplicate wells. After incubation for 24 hours, 10 μl MTT (500 mg/ml; Sigma-Aldrich) was added to the medium and cultured for another 4 hours. Then the medium was discarded and 150 μl dimethyl sulfoxide (Sigma-Aldrich) was added to dissolve the formation product. At last, the absorbance of each well was measured at a wavelength of 490 nm. Each assay was repeated three times at least.

### Flow cytometric analysis

The effect of TIPE2 on cell apoptosis was determined by FITC Annexin V staining followed by flow cytometric analysis. Cells were washed twice with cold PBS and resuspended in 1× binding buffer (BD Biosciences) at a concentration of 1 × 10^6^ cells/ml. 100 μl of the solution was transferred to a 5 ml culture tube, then 5 μl FITC Annexin V (BD Biosciences) and 10 μl PI (BD Biosciences) were added into the tube. After that, the cells were mixed gently and incubated for 15 minutes at room temperature in the dark. Finally, 1 ml PBS was added to each tube and samples were analyzed by flow cytometric within 1 hour. All experiments were repeated in triplicate.

### *In vivo* tumorigenesis assay

Six week old male BALB/c nude mice were purchased from Chinese Academy of Science (Shanghai, China) and maintained in specific pathogen-free conditions. 5 × 10^6^ H446 cells transfected with TIPE2 over-expressing vector (TIPE2 group) in 100 μl of PBS were injected subcutaneously into right flank while control vector (control group) into left flank of the nude mice, 10 nude mice were used in this experiment. Tumor size was measured every two days and the tumor volume was calculated according to the following formula: length × width × width × 0.5. About six weeks later, all the mice were sacrificed by cervical dislocation, then the tumors were removed, weighed, and fixed in formalin for the subsequent analysis.

### Statistical analysis

All statistical analyses were carried out using SPSS 13.0 software. The χ^2^ test was used for analysis of TIPE2 expression between lung cancer subtypes and adjacent non-tumor tissues. The student’s t-test was used for comparison among different groups. The differences in tumor growth and weight between two groups of nude mice were evaluated by repeated-measures analysis of variance. *P* < 0.05 was considered statistically significant.

## Results

### TIPE2 expression was down-regulated or lost in lung squamous cancer and small cell lung cancer tissues

**Fig 1:**
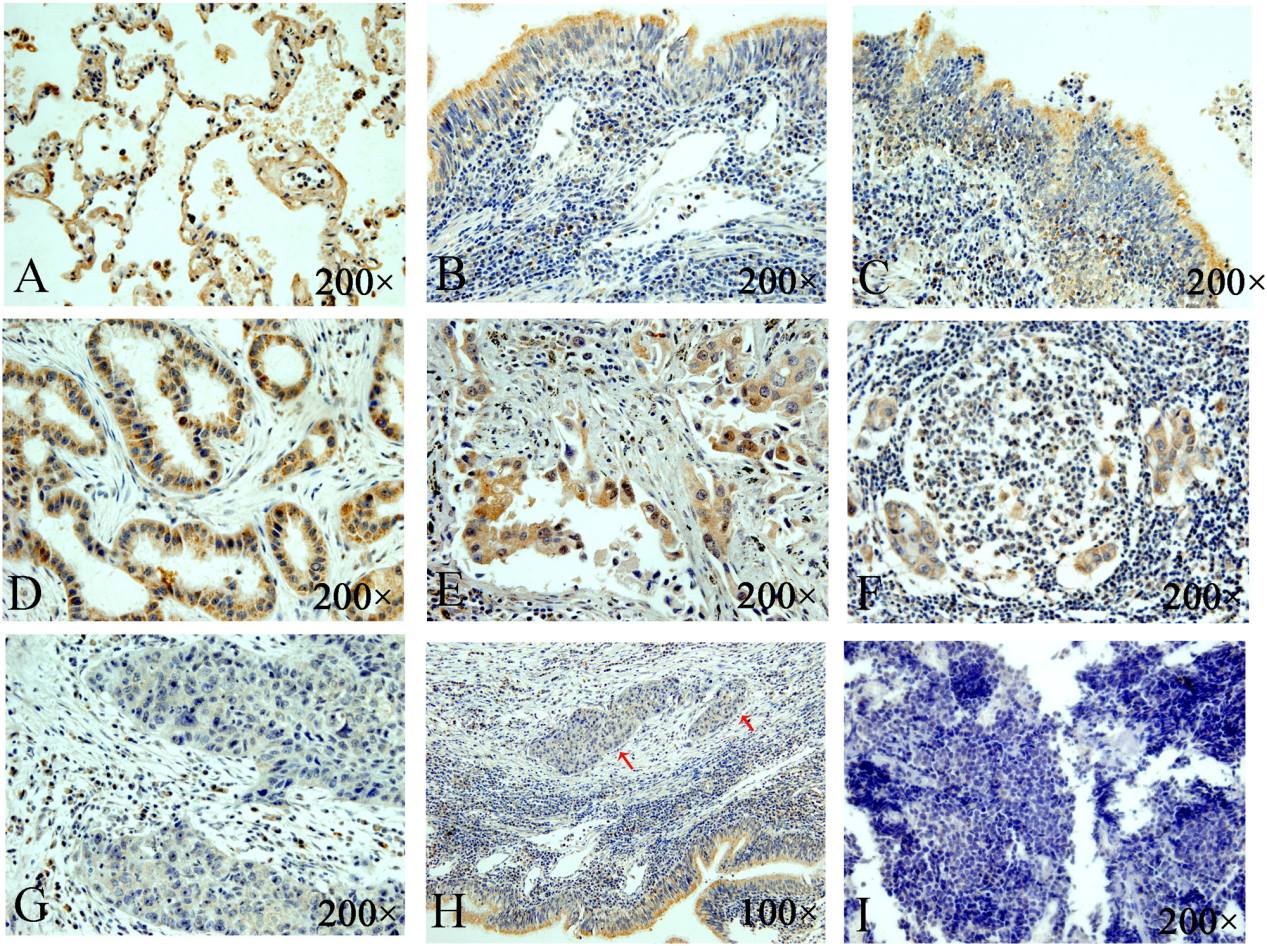
Immunohistochemistry analyzed TIPE2 expression in lung cancer tissues and adjacent non-tumor lung tissues. Representative pictures of TIPE2 expression in alveolar epithelium (A), bronchial epithelial (B), bronchial epithelial with squamous metaplasia (C), lung AdC (D, E), positive lymph node of lung AdC (F), lung squamous cancer (G, H) and small cell lung cancer (I).

Recent studies already revealed that TIPE2 expression were significantly correlated with some cancers such as RCC and HCC [15]. However, the TIPE2 expression pattern in lung cancer is unclear so far. To assess the relationship of TIPE2 expression and lung cancer tissues, we detected the expression status of TIPE2 in common histological types of lung cancer tissues by immunohistochemistry, including 25 lung adenocarcinoma tissues, 20 lung squamous carcinoma tissues, 15 small cell lung cancer tissues and 30 adjacent non-tumor lung tissues. As shown in (Fig 1A and 1B), in non-tumor lung tissues, TIPE2 strong staining was observed in lung epithelial alveolar cells and bronchial cells. And in bronchial epithelials with squamous metaplasia, TIPE2 still had strong staining (Fig 1C). In lung cancer tissues, TIPE2 showed strong staining in primary lung adenocarcinoma and lymph node metastasis tissue (Fig 1D, 1E and 1F), but exhibited very weak staining in lung squamous cancer and even almost no staining in most of small cell lung cancer (Fig 1G, 1H and 1I). Interestingly, (Fig 1H) showed very weak TIPE2 expression in the nests of lung squamous cancer cells (red arrows) and strong staining of TIPE2 in the adjacent bronchial epithelials simultaneously. In lung cancer stroma, no stain of TIPE2 was found in fibroblasts cells, but strong staining could be found in inflammatory cells such as plasmocytes and macrophagocytes. In positive cancer cells, TIPE2 staining was predominantly found in the cytoplasm but seldom in nuclear. Statistical analysis (Table 1) indicated TIPE2 expression was significantly down-regulated in lung squamous cancer and small cell lung cancer tissues compared to the non-tumor lung tissues, while no difference between lung adenocarcinoma and non-tumor lung tissues. The results demonstrated that TIPE2 was down-regulated in some histological subtypes of lung cancer, which encouraged us to further inquire into the specific role and underlying mechanism of TIPE2 in lung cancer development.

**Table 1:** The difference of TIPE2 between the subtypes of lung cancer and adjacent non-tumor lung tissues respectively.

tissues	N	Score		*P*
		low (0~3)	high (4~6)	
non-tumor lung tissues	30	5	25	
lung squamous cancer	25	20	5	0.000*
lung adenocarcinoma	20	1	19	0.179
small cell lung cancer	15	15	0	0.000*

* P<0.05 by χ2.

### TIPE2 inhibited the growth and promoted the apoptosis of lung cancer cell *in vitro*

**Fig 2:**
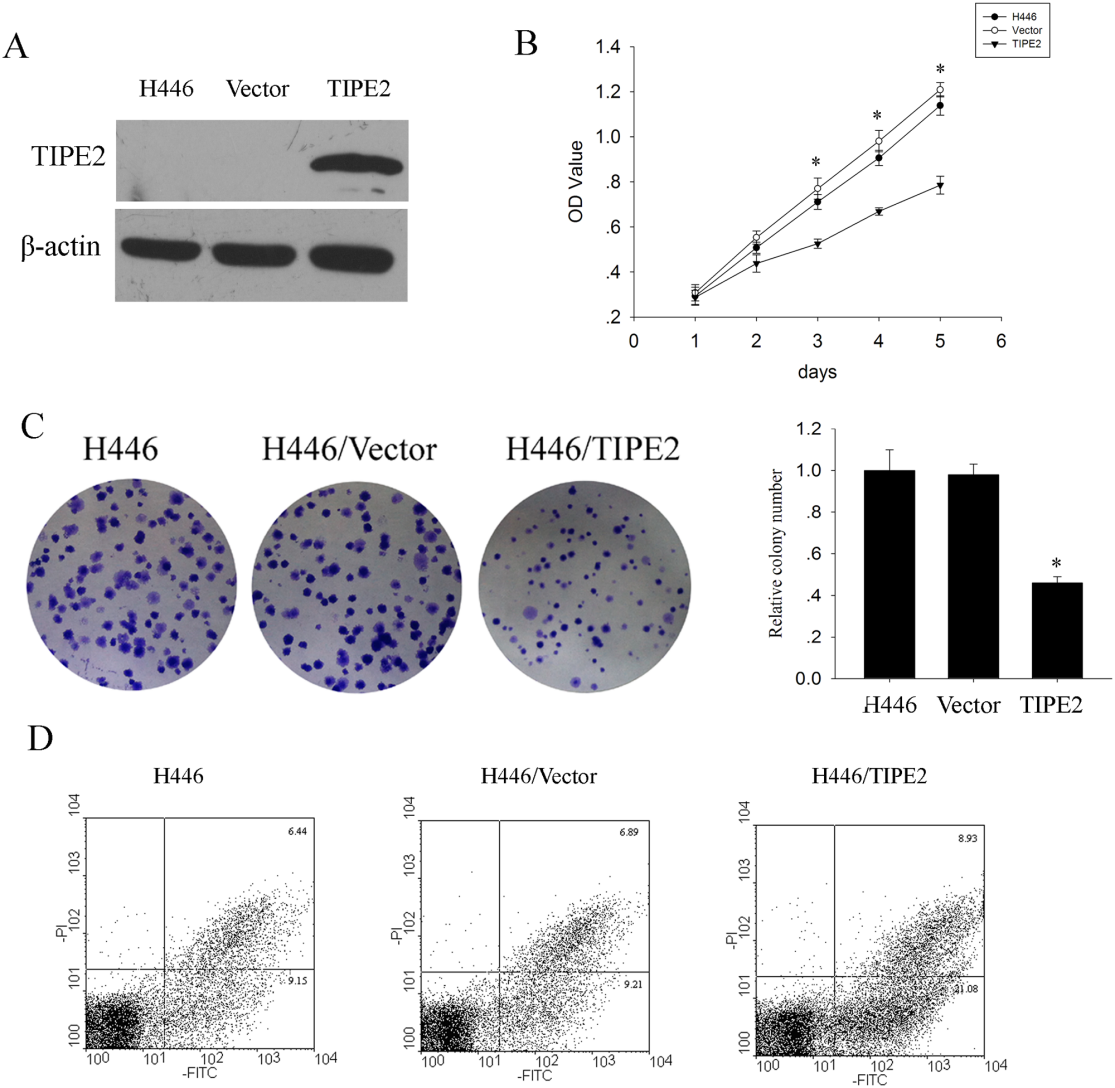
TIPE2 over-expression inhibited cell growth and promoted apoptosis. **(A), TIPE2 over-expression in H446 transfected with TIPE2 plasmid**. (B), Cell viability decreased after TIPE2 over-expression. (C), H446/TIPE2 had fewer colony formation compared to vector. (D), TIPE2 up-regulation increased the apoptotic rate significantly. * indicated *P* < 0.05.

To investigate the potential biological function of TIPE2 in lung cancer, we examined the effects of TIPE2 on cell growth of lung cancer cell H446 by MTT assay and colony formation assay, and apoptosis by flow cytometric. To up-regulate TIPE2 expression in H446, TIPE2 over-expressing vector was constructed and transfected stably in H446 cells. Western blotting validated the TIPE2 expression in H446 cells untransfected (H446/Null), control vector-transfected (H446/Control) and TIPE2 over-expressing vector transfected (H446/TIPE2). As shown in (Fig 2A), H446/Null and H446/Control showed almost no TIPE2 expression, while H446/TIPE2 revealed high level of TIPE2 expression. MTT assay (Fig 2B) and colony formation assay (Fig 2C) analysis showed reduction of cell viability and fewer colonies observed in H446/TIPE2 respectively, compared with H446/Control and H446/Null cells, which indicated TIPE2 could inhibit lung cancer cell growth. Cancer cell growth is affected by two main factors including proliferation and apoptosis. It was reported that murine TIPE2 could promote Fas-induced apoptosis [4]. Has TIPE2 potential role on apoptosis of lung cancer? Therefore, we applied flow cytometric to reveal effect of TIPE2 on apoptosis of H446. As shown in (Fig 2D), the early apoptosis rate of H446/TIPE2, H446/Control and H446/Null was 21.08%, 9.21% and 9.15% respectively, which showed over-expressing TIPE2 could significantly increase the apoptosis rate. These results suggested TIPE2 can effectively suppress the growth and promote the apoptosis of H446 cells *in vitro*.

### TIPE2 regulated Bcl-2/Bax balance, enhanced cleaved caspase-3 and caspase-9 levels

**Fig 3:**
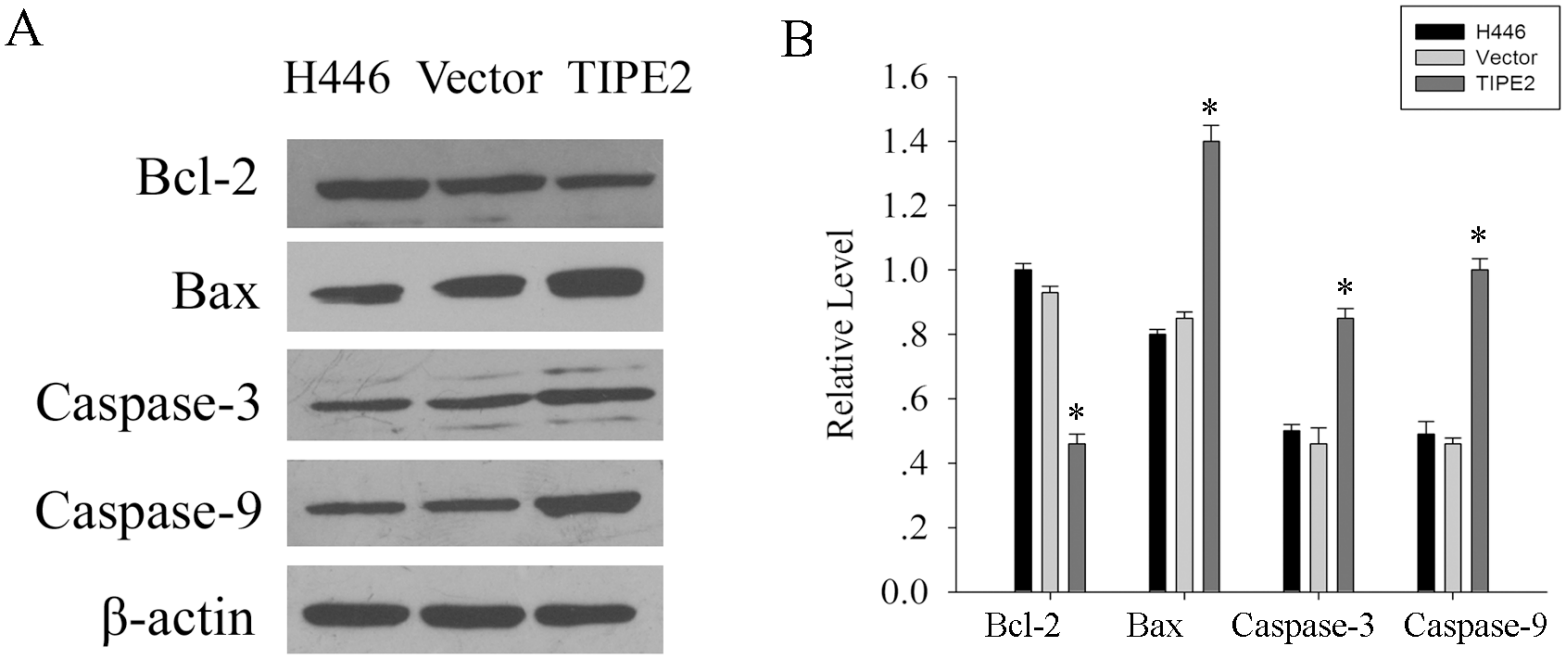
TIPE2 over-expression resulted in up-regulation of Bax, Caspase-3 and Caspase-9 and down-regulation of Bcl-2. (A), Western blot analysis of Bcl-2, Bax, Caspase-3 and Caspase-9 expression in H446, vector, TIPE2. (B), Histogram of the relative expression levels of the above-mentioned apoptosis-related molecules.

The results above-mentioned showed TIPE2 could promote apoptosis of H446 cells. Then, we were further interested in the underlying mechanism of it. It has been well known that Bcl-2/Bax balance plays an important role in cell apoptosis [19], so we investigated the effect of TIPE2 expression on Bcl-2/Bax balance. As shown in Fig 3, over-expressing TIPE2 could increase the Bax expression while decrease the Bcl-2 expression, which implied that the function of TIPE2 on cell apoptosis may be associated with Bcl-2/Bax balance. Furthermore, it has been demonstrated that caspase plays a critical role in executing the final pathway of apoptosis [20]. We further investigated whether TIPE2 would affected the activation of apoptotic molecular caspases such as caspase-3 and caspase-9. Therefore, we detected the expression of cleaved caspase-3 and caspase-9 in H446/Null, H446/Control and H446/TIPE2. As shown in Fig 3, TIPE2 enhanced the cleaved caspase-3 and caspase-9 expression levels, which suggested TIPE2 could promote the activation of caspase-3 and capase-9.

### TIPE2 increased activation of P38 MAPK pathway, while inhibiting activation of Akt

**Fig 4:**
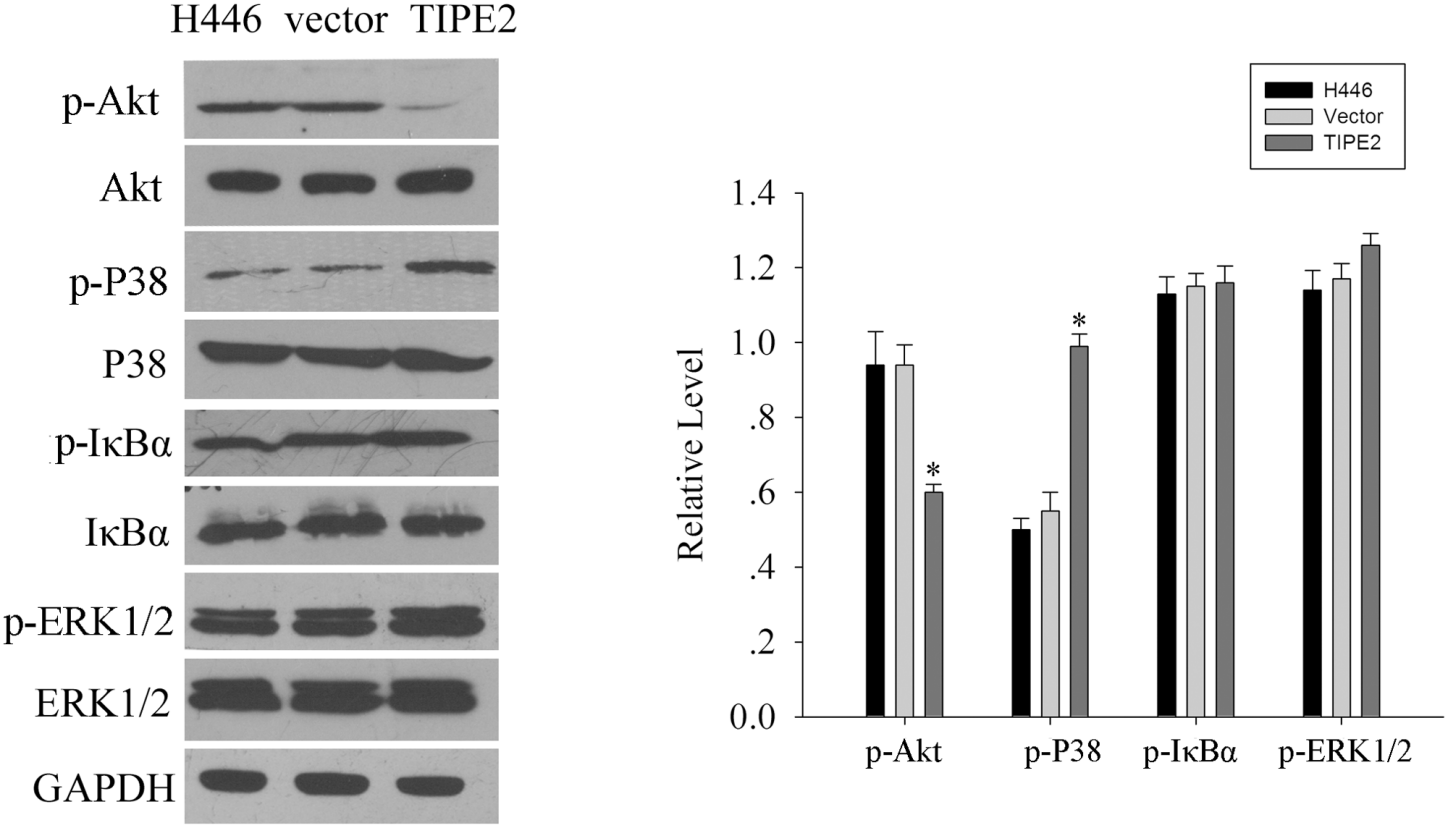
TIPE2 over-expression affected some signal molecules expression. (A), Western blot analysis of some common signal molecules expression in H446, vector, TIPE2. (B), Histogram of the relative expression levels of the above-mentioned signal molecules.

We also explored the effect of TIPE2 on several signaling pathways by analyzing key signaling molecules involved. We found that P38 and Akt pathway, but not ERK pathway and NF-κB pathway, were targets of TIPE2 action. High level expression of phosphorylated P38 MAPK was detected in H446 cells over-expressing TIPE2, while relatively low level expression in null and control cells (Fig 4), which indicated that TIPE2 may activate P38 MAPK pathway. However, the level of phosphorylated Akt was significantly decreased in H446 cells over-expressing TIPE2 compared to null and control cells (Fig 4). In addition, we also examined the expression of other signal molecules such as phospho-ERK, phospho-MEK, and phospho-IκBα in H446/Null, H446/Control and H446/TIPE2, among which little or no difference was noted (Fig 4). Taken together, these data indicated that TIPE2 could regulate P38 and Akt pathway, in which TIPE2 was a positive regulator of P38 pathway while negative regulator of Akt pathway.

### TIPE2 obviously inhibited tumor formation *in vivo*

**Fig 5:**
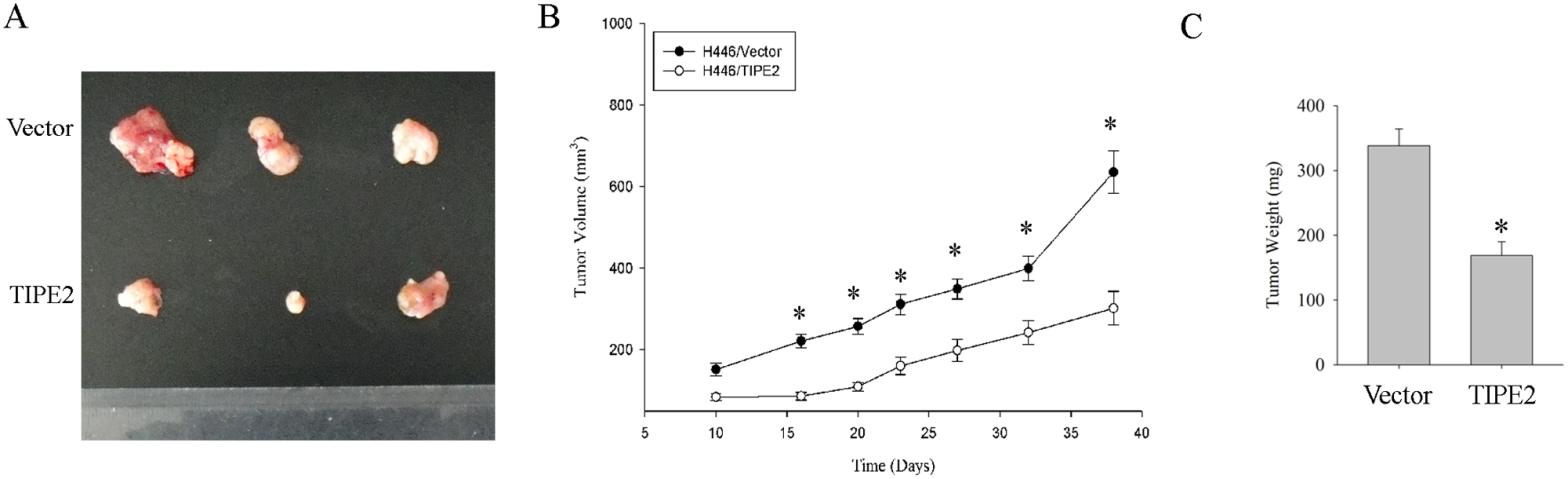
Over-expression of TIPE2 suppressed tumor formation. (A), A representative picture of the isolated tumors. (B), Subcutaneous tumor growth curve of nude mice injected with H446/vector and H446/TIPE2. (C), The mean tumor weights of vector and TIPE2 group. * indicated *P* < 0.05.

**Fig 6:**
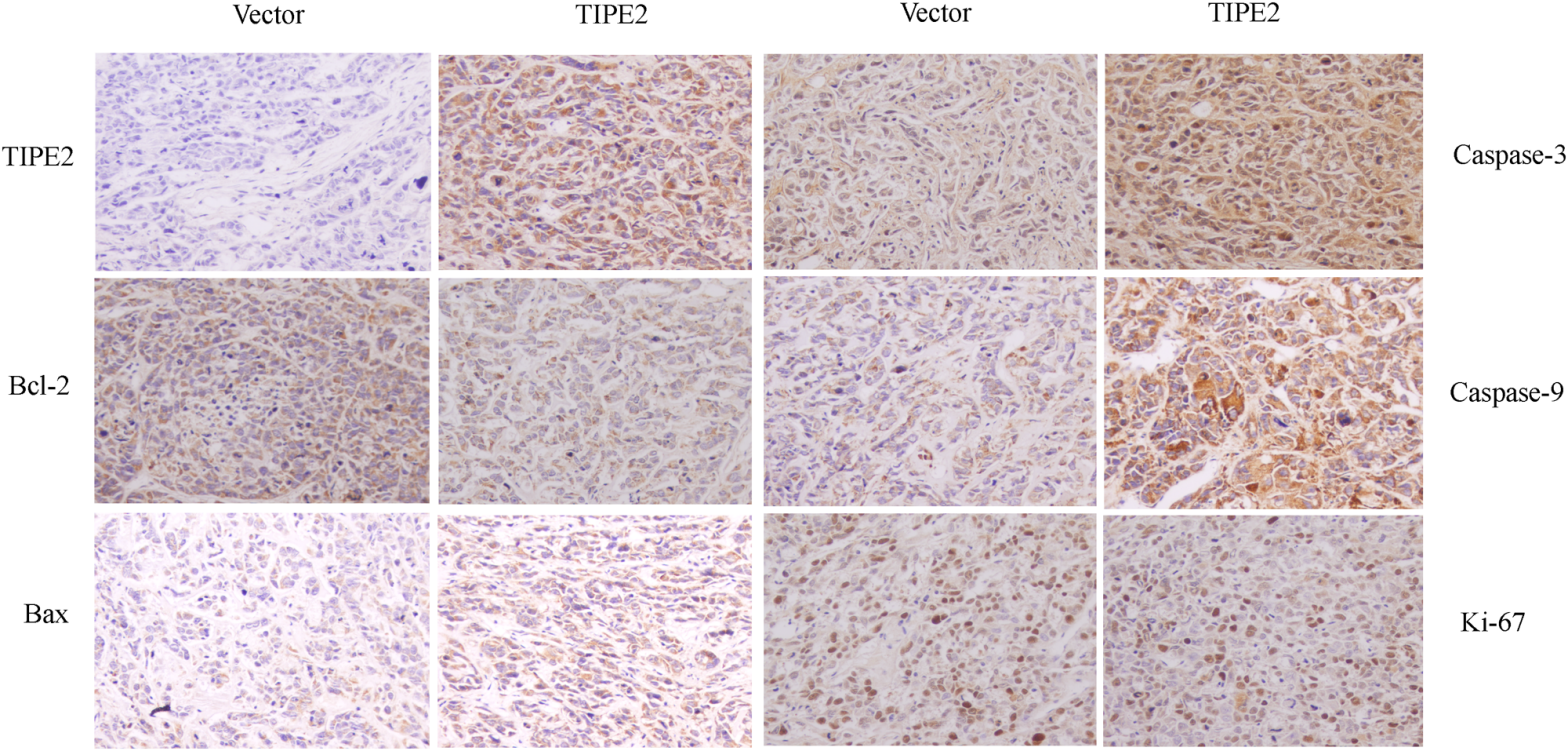
Immunohistochemistry detected TIPE2, Bcl-2, Bax, Caspase-3, Caspase-9 and Ki-67 expression in the isolated subcutaneous tumor tissues from vector and TIPE2 group. Magification, 200 ×.

The above-mentioned results showed TIPE2 could effectively suppress the growth and promote the apoptosis of H446 cells *in vitro*. Next, we investigated its effect on tumorigenesis *in vivo*. H446 cells transfected with control vector or TIPE2-expressing vector were injected subcutaneously into nude mice to initiate tumor formation. From the curve of tumor growth (Fig 5B), the tumor volume of TIPE2-expression groups were significantly decreased compare to the vector groups. About six weeks later, all groups of nude mice were sacrificed and the tumors were isolated and weighted (Fig 5A). The overall mean tumor size of TIPE2-expression groups was significantly smaller than that of the vector groups, which was consistent with the tumor weights (Fig 5C). All these results demonstrated that overexpression of TIPE2 could inhibit the subcutaneous tumor growth *in vivo* obviously. As above-mentioned, over-expression of TIPE2 had significant impacts on the expression of some apoptotic molecules in H446 cells *in vitro*, such as Bcl-2, Bax, caspase-3 and caspase-9. So, it is necessary to elucidate the effect of TIPE2 on these molecules expression in the tumors from nude mice *in vivo*. The tumors from nude mice were isolated, fixed, embedded and made into sections, which were then stained by immunohistochemistrical analysis. Results showed overexpression of TIPE2 while a increased expression of Bax, caspase-3 and caspase-9 and a reduced expression of Bcl-2 in TIPE2-overexpression tumors (Fig 6), which were similar to the results *in vitro*. We also detected the expression of Ki-67, a proliferation marker, in above tumors. No alteration on Ki-67 expression was detected regardless of TIPE2 over-expression (Fig 6), by which we speculated that TIPE2 inhibits tumor growth mainly attributing to its function of promoting apoptosis of H446 cells, but not via affecting proliferation.

## Discussion

TIPE2 belongs to TIPE (TNFAIP8) family, which is a recently identified group of proteins including four members, TNFAIP8, TIPE1, TIPE2 and TIPE3 [4]. TNFAIP8 is associated with enhanced cell survival and inhibition of apoptosis [21]. Knocking down the expression of TNFAIP8 in tumor cells can reduce their tumorigenicity [22]. All these evidences support that TIPE is an oncogene and it may be linked with the cell survival and malignant growth-related signaling pathways [11]. High level of TIPE1 mRNA is detected in most human carcinoma cell lines, which suggests that it may play a role in carcinogenesis [11]. Unlike TNFAIP8 and TIPE1, TIPE2 was mainly found played a vital role in immunity. As reported, TIPE2 was a novel gene which maintains immune homeostasis and negatively regulates innate and adaptive immunity [4]. However, information about TIPE2 in human cancer is limited.

A few studies on the relationship of TIPE2 with cancer already verified TIPE2 had some function in cancer development. In recent research, TIPE2 was thought as a tumor suppressor in hepatocellular carcinoma [16]. However, the relationship between TIPE2 and lung cancer is unknown so far. In our present study, we demonstrated the state of TIPE2 expression in lung cancer. We found that TIPE2 expression was down-regulated in lung squamous carcinoma and even almost lost in small cell lung cancer. However, in lung adenocarcinoma, TIPE2 strong expression was found, but no difference between non-tumor lung tissues and lung adenocarcinoma was analyzed. These results showed TIPE2 expression patterns might be different among different histologic subtypes of lung cancer, which suggested TIPE2 might play some different biological roles in different histologic types of lung cancer. To find out TIPE2 expression in all kinds of lung cancer, increaseing the numbers of lung cancer samples in the future study may be necessary.

Furthermore, this study preliminarily interpreted the biological function of TIPE2 in lung cancer. Over-expressing TIPE2 promoted apoptosis of H446 cells *in vitro* and inhibited tumor growth *in vivo*. At the molecular level, TIPE2 regulated Bcl-2/Bax balance and activated caspase-3 and caspase-9.

Interested in looking into the specific signal molecules possibly regulated by TIPE2, we then screened the expression of some common signaling molecules after TIPE2 over-expression. High level expression of phosphorylated P38 MAPK was detected after TIPE2 up-regulation. Recent studies have suggested a key role for P38 MAPK in mediating pathways leading to apoptosis and growth inhibitory signals [23,24]. Additionally, P38 MAPK triggers the activation of caspase-3, 9 and is also necessary for the phosphorylation of apoptosis-related proteins, including Bax, Bim and Bcl-2 in OA-treated cancer cells [25]. Combined with these results, we speculate that TIPE2 promotes lung cancer cell apoptosis through activation of P38 MAPK, which triggers the over-expression of caspase-3 and caspase-9. In addition, we also found that TIPE2 decreased the phosphorylated level of Akt. As reported, the Akt pathway is a central signal transduction pathway that regulates many critical aspects of cancer physiology, including cell proliferation, cell morphology, migration and apoptosis [26]. Meanwhile, some apoptosis-related molecules such as Bcl-2, Bax, caspase-3 and caspase-9 are affected distinctly by TIPE2. So, our results provide a lot of evidence that TIPE2 promotes lung cancer apoptosis. Interestingly, we found Ki-67 expression had no alteration after TIPE2 over-expression, which indicated that TIPE2 inhibited lung cancer growth attributing to promotion of cell apoptosis.

Taken together, TIPE2 functions as a tumor suppressor to promote lung cancer cell apoptosis. Therefore, the forced expression of human TIPE2 may be a new strategy for treatment of lung cancer.
